# Dynamical transmission model of MERS-CoV in two areas

**DOI:** 10.1063/1.4942993

**Published:** 2016-02-29

**Authors:** Benny Yong, Livia Owen

**Affiliations:** 1,2 Department of Mathematics, Faculty of Information Technology and Science, Parahyangan Catholic University, Jalan Ciumbuleuit 94 Bandung 40141, West Java, INDONESIA; Institut Teknologi Bandung, Bandung, Indonesia; Institut Teknologi Bandung, Bandung, Indonesia

## Abstract

Middle East Respiratory Syndrome Coronavirus (MERS-CoV) is a disease first reported in Saudi Arabia in 2012 and it can be transmitted from human to human. This disease has spread to several other countries, most confirmed cases have displayed symptoms of severe acute respiratory illness and many of these patients have died. This research is aimed to construct a mathematical model for the transmission of MERS-CoV in two areas by separating the human population into two groups; susceptible and infectious groups. The dynamics of the disease is studied by a compartmental model involving ordinary differrential equations. The basic reproductive number of this disease is discussed to control the outbreak of this disease. Sensitivity analysis of this model is performed to determine the relative importance of the model parameters to the MERS-CoV transmission.

## INTRODUCTION

In recent years, mathematical models are increasingly used by researchers to understand the transmission of infectious diseases (H. W. Hethcote, 2000). Many models for the spread of infectious diseases in populations have been analyzed mathematically and applied to specific diseases (Z. Ma and J. Li, 2009). Mathematical modelling plays a keyrole in policy making, including risk assessment and control programme evaluation in reducing morbidity and mortality (N. Chitnis, *et al.*, 2008).

Middle East respiratory syndrome coronavirus (MERS-CoV), previously known as novel coronavirus was first identified in humans in 2012. It can cause severe acute respiratory disease, particularly in people with underlying conditions. The MERS-CoV is a potential pandemic disease, cases of this disease has been reported in some countries. As of 11 September 2015, World Health Organization (WHO) global case count was 1,569 laboratory-confirmed cases of MERS-COV, including at least 554 deaths (case fatality rate 35.31%) since the first cases were reported in September 2012 (WHO, 2015). All cases have had a history of residence in or travel to the Middle East (>90% Saudi Arabia), or contact with travellers returning from these areas (L. M. Gardner and C. R. MacIntyre, 2014). Until now, there is no vaccine for this disease.

Mathematical modeling for disease transmission has been done by many different authors to understand the dynamical spread of disease in humans, for example in S. Syafruddin and M. S. M. Noorani (2011), B. Yong (2007), and Z. Feng, *et al.* (2000). Models for infectious disease are helpful for prevention and control of emerging infectious disease like MERS-CoV. Here a SISI (S for susceptible and I for infectious) epidemiological model for human to human in two areas describing MERS-CoV disease transmission is presented, as well as the associated basic reproductive number. Firstly, we formulate a SISI model to describe the transmission of MERS-CoV in two areas. Next, we evaluate the basic reproductive number using the next generation matrix method. Basic reproductive number is discussed in order to identify influential model parameters, so with controlling parameters in it, the outbreak of the disease can be eliminated. Finally, we analyze sensitivity of the model in order to determine the influence of the input parameters on the model outputs. Based on this analysis, we can find which parameters are most sensitive to the MERS-CoV transmission model.

## MATHEMATICAL MODEL

The model describes the dynamic of MERS-CoV transmission. We divide the population ($N$) into two areas, namely area $x$ and $y$. In each area, we have two sub-populations, according to their disease status; population who are susceptible to infection ($S_x$ and $S_y$) and population who have the disease ($I_x$ and $I_y$). Initially, there are susceptible and infectious humans in each area. Individuals are born into the susceptible class and individuals susceptible to infection. There is a natural death rate of human population from each compartment and its value is same in both areas of population. Someone who gets infected and then recovers will return to the susceptible class. The susceptible population in area $x$ ($S_x$) is increased by recruitment of individuals $a_1$, susceptible individuals from area $y$ leave to area $x$ with rate $\alpha_2$, and infected individuals in area $x$ recover with rate $d$. This population is reduced through infection

*Application of Mathematics in Industry and Life*
AIP Conf. Proc. 1716, 020010-1–020010-7; doi: 10.1063/1.4942993
© 2016 AIP Publishing LLC 978-0-7354-1363-4/$30.00

within area $x$ with transmission rate $\beta$ (moving to class $I_x$), susceptible individuals from area $x$ leave to area $y$ with rate $\alpha_1$ (moving to class $S_y$), individuals from area $y$ leave to area $x$ and they infected with transmission rate $\omega\alpha_2$, and by natural death with rate $b$. The population of infectious individuals is increased by infection of susceptible within area $x$ with transmission rate $\beta$, infected individuals from area $y$ leave to area $x$ with rate $\alpha_2$, and individuals from area $y$ leave to area $x$ and they infected with transmission rate $\omega\alpha_2$. It is diminished by death due to disease with rate $c$, by recovery from the disease with rate $d$ (moving to class $S_x$), and infected individuals from area $x$ leave to area $y$ with rate $\alpha_1$ (moving to class $I_y$).

Meanwhile, the susceptible population in area $y$ ($S_y$) is increased by recruitment of individuals $a_2$, susceptible individuals from area $x$ leave to area $y$ with rate $\alpha_1$, and infected individuals in area $y$ recover with rate $d$. This population is reduced through infection within area $y$ with transmission rate $\beta$ (moving to class $I_y$), susceptible individuals from area $y$ leave to area $x$ with rate $\alpha_2$ (moving to class $S_x$), individuals from area $x$ leave to area $y$ and they infected with transmission rate $\omega\alpha_1$, and by natural death with rate $b$. The population of infectious individuals is increased by infection of susceptible within area $y$ with transmission rate $\beta$, infected individuals from area $x$ leave to area $y$ with rate $\alpha_1$, and individuals from area $x$ leave to area $y$ and they infected with transmission rate $\omega\alpha_1$. It is diminished by death due to disease with rate $c$, by recovery from the disease with rate $d$ (moving to class $S_y$), and infected individuals from area $y$ leave to area $x$ with rate $\alpha_2$ (moving to class $I_x$).

The detailed transition between these four compartments is depicted in Fig. 1.

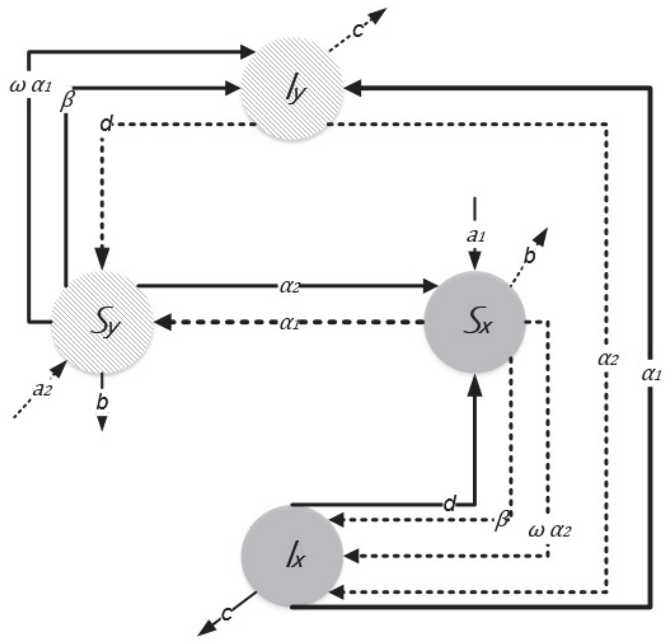

**FIGURE 1. A transmission diagram of the SISI MERS-CoV model in two areas**

With the assumptions given and the illustrations in Fig. 1, we obtain the following four-dimensional system of nonlinear differential equation for the MERS-CoV transmission:

$$\left.\begin{aligned}
\frac{dI_x(t)}{dt} &= \frac{\beta\, S_x I_x}{S_x + I_x} - (c + d + \alpha_1)\, I_x + \alpha_2\, I_y + \frac{\omega\, \alpha_2\, S_y\, I_y}{S_y + I_y} \\
\frac{dI_y(t)}{dt} &= \frac{\beta\, S_y I_y}{S_y + I_y} - (c + d + \alpha_2)\, I_y + \alpha_1\, I_x + \frac{\omega\, \alpha_1 S_x\, I_x}{S_x + I_x} \\
\frac{dS_x(t)}{dt} &= a_1 - \frac{\beta\, S_x I_x}{S_x + I_x} - (b + \alpha_1)\, S_x + \alpha_2\, S_y + d\, I_x - \frac{\omega\, \alpha_2\, S_y\, I_y}{S_y + I_y} \\
\frac{dS_y(t)}{dt} &= a_2 - \frac{\beta\, S_y\, I_y}{S_y + I_y} - (b + \alpha_2)\, S_y + \alpha_1\, S_x + d\, I_y - \frac{\omega\, \alpha_1 S_x\, I_x}{S_x + I_x}
\end{aligned}\right\} \quad (1)$$

The variable domain of the model is

$$\Omega = \{(I_x, I_y, S_x, S_y) \in \mathbb{R}^4 : I_x, I_y, S_x, S_y \geq 0\}$$

and all parameters used in the model; $a_1, a_2, b, c, d, \beta, \alpha_1, \alpha_2$, and $\omega$ are positive. It can be verified that $\Omega$ is a positively invariant set with respect to model.

**TABLE 1. Parameters used in Fig. 1 and their description**

| Description | Parameter |
|---|---|
| Number of newly recruited to the susceptible $\boldsymbol{x}$ population | $a_1$ |
| Number of newly recruited to the susceptible $\boldsymbol{y}$ population | $a_2$ |
| Natural death rate for susceptible individuals | $b$ |
| MERS-CoV death rate of human population | $c$ |
| Recovery rate from MERS-CoV | $d$ |
| Transmission rate within an area | $\beta$ |
| Movement rate of human population from area $\boldsymbol{x}$ leave to area $\boldsymbol{y}$ | $\alpha_1$ |
| Movement rate of human population from area $\boldsymbol{y}$ leave to area $\boldsymbol{x}$ | $\alpha_2$ |
| Transmission rate in different area | $\omega$ |

The model (1) has two equilibrium points which are given by $E_0 = (I_x{}^*, I_y{}^*, S_x{}^*, S_y{}^*) = \left(0, 0, \frac{a_1\alpha_2 + a_2\alpha_2 + a_1 b}{b\,(\alpha_1+\alpha_2+b)}, \frac{a_1\alpha_1 + a_2\alpha_1 + a_2 b}{b\,(\alpha_1+\alpha_2+b)}\right)$ and $E_1 = (I_x{}^{**}, I_y{}^{**}, S_x{}^{**}, S_y{}^{**})$. Equilibrium point $E_0$ represents the situation where only $S_x{}^*$ and $S_y{}^*$ exist and it is called disease free equilibrium (DFE) point whereas equilibrium point $E_1$ depicts the situation where all population exist and it is called endemic equilibrium (EE) point.

## BASIC REPRODUCTIVE NUMBER

Basic reproductive number is an important threshold in mathematical epidemiology. This threshold conditions determine whether an infectious disease will spread in a susceptible population when the disease is introduced into the population (O. Diekmann and J. A. P. Heesterbeek, 2000). The threshold is calculated by using the spectral radius of a next generation (infection) matrix of a model (P. van den Driessche and J. Watmough, 2002). It is given mathematically as

$$R_0 = \rho(FV^{-1})$$

where $\rho$ is defined as the spectral radius of the next generation matrix $FV^{-1}$, $F$ is the rate of appearance of new infections in compartment $i$, and $V$ is the transfer of individuals out of compartment $i$ by all other means.

Given the DFE $E_0$. Basic reproductive number $R_0$ is calculated as the largest eigenvalue (spectral radius) of the matrix of partial derivatives (Z. Ma and J. Li, 2009):

$$F = \left[\frac{\partial \mathcal{F}_i(E_0)}{\partial x_j}\right] = \begin{bmatrix} \beta & \omega\alpha_2 \\ \omega\alpha_1 & \beta \end{bmatrix}$$

and

$$V = \left[\frac{\partial \mathcal{V}_i(E_0)}{\partial x_j}\right] = \begin{bmatrix} c + d + \alpha_1 & -\alpha_2 \\ -\alpha_1 & c + d + \alpha_2 \end{bmatrix}$$

where

$$\mathcal{F}_i(x) = \begin{bmatrix} \dfrac{\beta S_x I_x}{S_x + I_x} + \dfrac{\omega\alpha_2 S_y I_y}{S_y + I_y} \\ \dfrac{\beta S_y I_y}{S_y + I_y} + \dfrac{\omega\alpha_1 S_x I_x}{S_x + I_x} \\ d\, I_x \\ d\, I_y \end{bmatrix}, \qquad \mathcal{V}_i(x) = \begin{bmatrix} (c + d + \alpha_1) I_x - \alpha_2 I_y \\ (c + d + \alpha_2) I_y - \alpha_1 I_x \\ -a_1 + \dfrac{\beta S_x I_x}{S_x + I_x} + bS_x + \alpha_1 S_x - \alpha_2 S_y + \dfrac{\omega\alpha_2 S_y I_y}{S_y + I_y} \\ -a_2 + \dfrac{\beta S_y I_y}{S_y + I_y} + bS_y + \alpha_2 S_y - \alpha_1 S_x + \dfrac{\omega\alpha_1 S_x I_x}{S_x + I_x} \end{bmatrix}$$

Therefore, the next generation matrix is given as follows

$$FV^{-1} = \begin{bmatrix} \dfrac{\alpha_2(\alpha_1\omega + \beta) + \beta(c+d)}{(c+d)(c+d+\alpha_1+\alpha_2)} & \dfrac{\alpha_2(\beta + \omega(c+d+\alpha_1))}{(c+d)(c+d+\alpha_1+\alpha_2)} \\ \dfrac{\alpha_1(\beta + \omega(c+d+\alpha_2))}{(c+d)(c+d+\alpha_1+\alpha_2)} & \dfrac{\alpha_1(\alpha_2\omega + \beta) + \beta(c+d)}{(c+d)(c+d+\alpha_1+\alpha_2)} \end{bmatrix}$$

The spectral radius of the next generation matrix is

$$R_0 = \frac{\beta(\alpha_1 + \alpha_2 + 2c + 2d) + 2\alpha_1\alpha_2\omega + \sqrt{\rho}}{2(\alpha_2 + \alpha_1)(c+d) + (c+d)^2} \tag{2}$$

with

$$\rho = \beta^2(\alpha_1 + \alpha_2)^2 + 4\alpha_1\alpha_2\omega\left((c+d+\alpha_2)(c+d+\alpha_1)\omega + 2\beta\left(\frac{1}{2}\alpha_1 + \frac{1}{2}\alpha_2 + c + d\right)\right)$$

As shown in (2), the basic reproductive number of system (1) depends on parameters $\beta, \alpha_1, \alpha_2, c, d,$ and $\omega$. Equilibrium point $E_0$ will be locally asymptotic stable iff $R_0 < 1$. It is easily verified that all eigenvalues are negative at this point. Meanwhile, equilibrium point $E_1$ exist iff $R_0 > 1$.

In this paper, we use parameter values $\alpha_1 = 4{,}326, \alpha_2 = 13{,}461, b = 0.01, c = 0.05, d = 0.1, \beta = 0.1,$ and $\omega = 0.08$. As described in Fig. 2 and Fig. 3, the bigger the movement rate of human population ($\alpha_1$ and $\alpha_2$), the larger the rate of $R_0$.

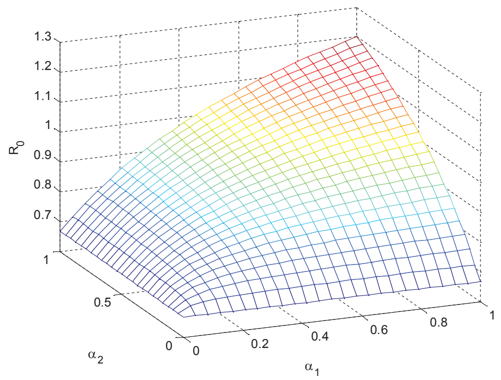

**FIGURE 2. Plot of $R_0$ for variation $\alpha_1$ and $\alpha_2$**

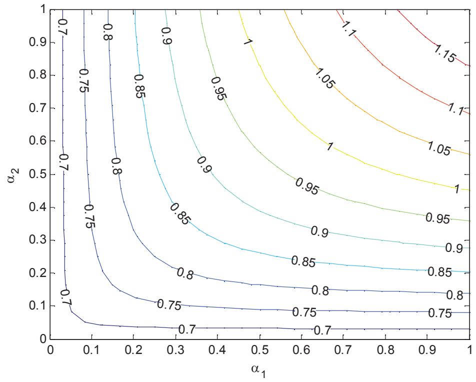

**FIGURE 3. Level set of $\alpha_1$ and $\alpha_2$ with respect to $R_0$**

In the next section, the sensitivity indices of $R_0$ related to the parameters in the model are calculated. Sensitivity indices allow us to measure the relative change in a variable when a parameter changes.

## SENSITIVITY ANALYSIS

Since learning about the influence of the parameters on the behavior of the model is of much interest, it is critical to carry out a sensitivity analysis. The main goal of this section is to perform sensitivity analysis of MERS-CoV transmission model to the parameters describing it, i.e. to determine the amount that the entire model changes when each parameter is altered. Sensitivity analysis is often used to study how the variation in the output of a model can be apportioned, qualitatively or quantitavely, to different sources of variation, and of how the given model depends on the information fed into it (A. Saltelli, *et al.*, 2000). Sensitivity analysis allows us to assess the impact that changes in a certain parameter will have on the model and it can help someone to determine which parameters are the key drivers of a model's results.

The sensitivity index of the basic reproductive number with respect to the parameter $p$ is given as follows

$$SI_{R_0} = \frac{\frac{\partial R_0}{R_0}}{\frac{\partial p}{p}} = \frac{\partial R_0}{\partial p} \times \frac{p}{R_0}$$

Here we give two cases for sensitivity indices of $R_0$; $R_0 < 1$ and $R_0 > 1$. As shown in Table 2, parameter $\beta$ gives the biggest positive effect on the change of $R_0$ than other parameters.

**TABLE 2. Sensitivity indices of $R_0$**

| Parameter | $SI_{R_0<1}(\alpha_1 = 0.6, \alpha_2 = 0.2)$ | $SI_{R_0>1}(\alpha_1 = 0.8, \alpha_2 = 0.6)$ |
|---|---|---|
| $c$ | $-0.3326598246$ | $-0.3332527390$ |
| $d$ | $-0.6653196494$ | $-0.6665054780$ |
| $\beta$ | +0.8030733632 | +0.6454323343 |
| $\alpha_1$ | +0.0520516272 | +0.1536403789 |
| $\alpha_2$ | +0.1428544841 | +0.2006855042 |
| $\omega$ | +0.1969266369 | +0.3545676659 |

In Fig. 4, we show effects on the number of infected humans through parameters variation for condition $R_0 < 1$.

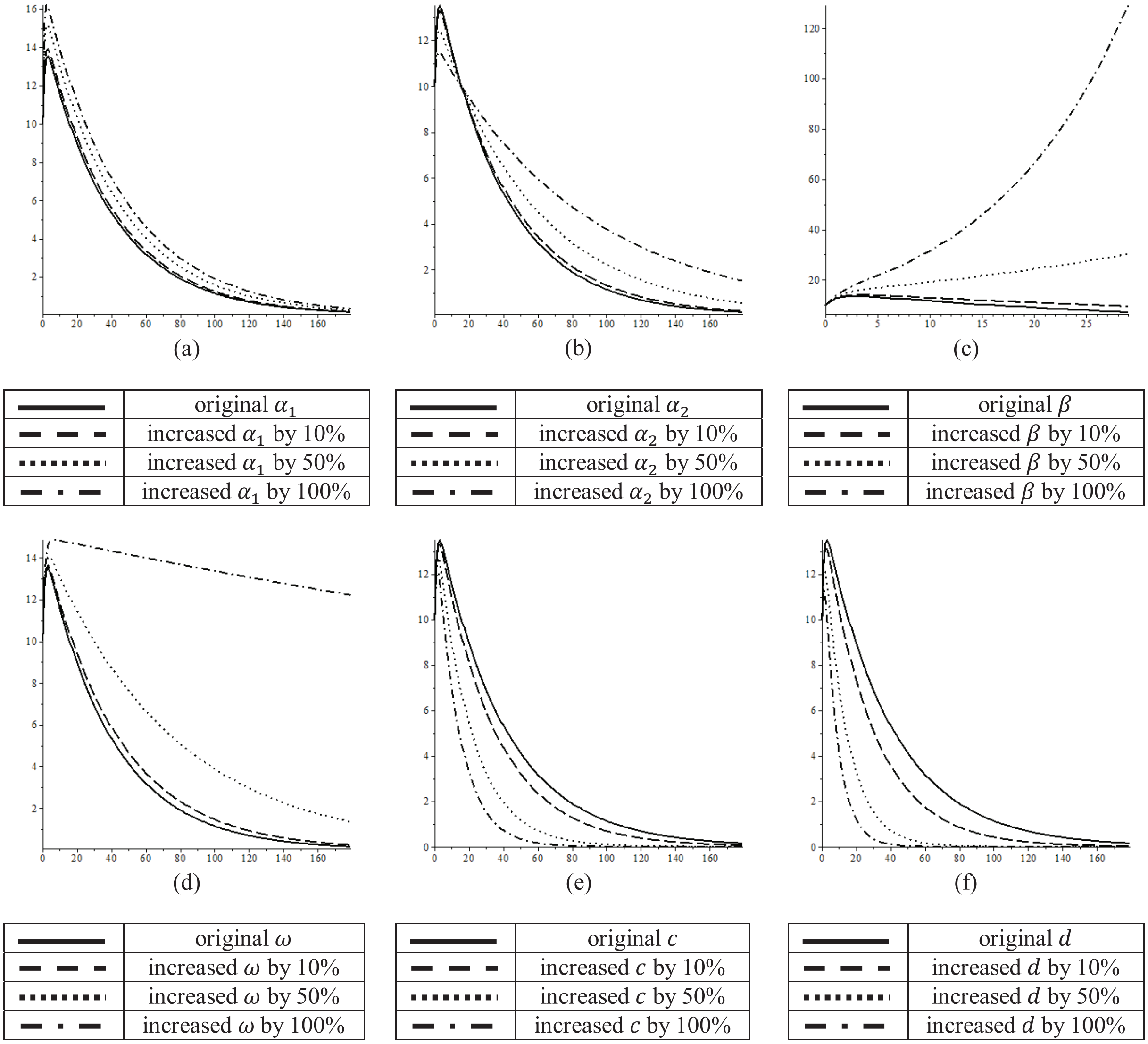

**FIGURE 4. Effect on $I_y$ along the time $t$ (in weeks) of the variation of $\alpha_1$ (a), $\alpha_2$ (b), $\beta$ (c), $\omega$ (d), $c$ (e), and $d$ (f) with $\alpha_1 = 0.6, \alpha_2 = 0.2$ ($R_0 < 1$)**

In Fig. 5, we show effects on the number of infected humans through parameters variation for condition $R_0 > 1$.

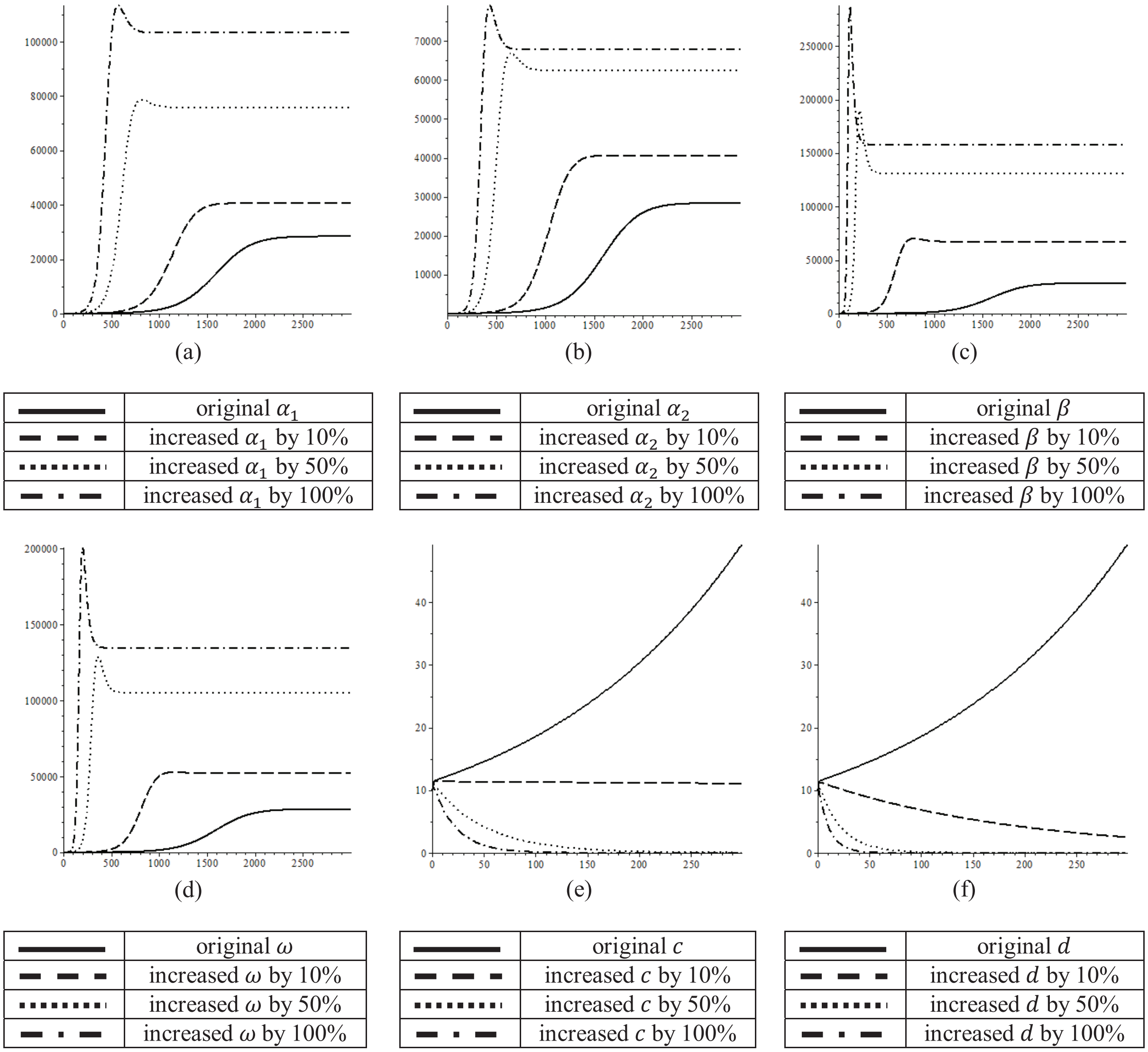

**FIGURE 5. Effect on $I_y$ along the time $t$ (in weeks) of the variation of $\alpha_1$ (a), $\alpha_2$ (b), $\beta$ (c), $\omega$ (d), $c$ (e), and $d$ (f) with $\alpha_1 = 0.8, \alpha_2 = 0.6$** ($R_0 > 1$)

In both figures (Fig. 4 and Fig. 5), it can be seen that parameters $c$ and $d$ have a negative sign in the sensitivity indices of $R_0$, while parameters $\beta, \alpha_1, \alpha_2$, and $\omega$ have a positive sign in the sensitivity indices of $R_0$.

## CONCLUSION

This paper discusses about dynamical transmission model of MERS-CoV in two areas. The model has two equilibrium points, disease free equilibrium point $E_0$ and endemic equilibrium point $E_1$. The disease dies out if the basic reproductive number is less than unity and the disease is established in the population if the basic reproductive number is greater than unity. It can be seen from basic reproductive number that MERS-CoV transmission model in two areas depends on parameters $\beta, \alpha_1, \alpha_2, c, d$, and $\omega$. From the sensitivity indices, the number of infected humans can be reduced by increasing $c$ and $d$ and/or decreasing $\beta, \alpha_1, \alpha_2$, and $\omega$. We can see that $\beta$ is the most positive sensitive parameter in the model. With controlling this parameter continuously, the number of infected humans can be decreased significantly.

## ACKNOWLEDGEMENTS

The authors would like to thank UNPAR Monodisiplin Research Grant 2015.
